# At the forefront of the sequencing revolution—notes from the RNGS19 conference

**DOI:** 10.1186/s13059-019-1714-3

**Published:** 2019-05-30

**Authors:** Sander Wuyts, Nicola Segata

**Affiliations:** 1 Department of Bioscience Engineering, University of Antwerp, Antwerp, Belgium; 2 Department CIBIO, University of Trento, Trento, Italy

## Abstract

Herein we present a meeting report on the third edition of the ‘Revolutionizing Next-Generation Sequencing’ conference, organized by the Flemish life-science research institute VIB and held at Antwerp, Belgium, 25–26 March 2019.

## Introduction

Next-generation sequencing (NGS) has been a game changer for many disciplines in biology for almost two decades. When it was in its early phases, NGS focused on increasing the throughput of the sequencing process, but this technology is now undergoing ‘differentiation’ to serve more-specific applications, including single-cell genomics, epigenomics, environmental ‘metagenomics’, and clinical real-time diagnostics. NGS techniques have not yet met their full revolutionary potential, and this meeting, the third edition of the ‘Revolutionizing Next-Generation Sequencing’ conference held in Antwerp, Belgium, aimed at fostering the academic and commercial debate around this suite of technologies. With 17 academic speakers presenting their work on fields of diverse application, ranging from the dynamics of plankton populations drifting in the oceans to the first genome sequences of multiple bat species, together with 15 industry speakers showcasing their newest developments, this get-together was indeed at the forefront of the NGS revolution. Here, we give a concise overview of how the community has spent the past few years revolutionizing NGS. We highlight some representative talks and sessions—without attempting to cover the whole program owing to space constraints.

## Long-read sequencing

Nick Loman (University of Birmingham, UK) kicked-off the meeting with a hands-on talk about the potential of long-read sequencing technologies (Oxford Nanopore Technologies and Pacific Biosciences) in multiple scientific fields. If NGS sequencing is questioning the hypothesis-driven method with the temptation of a ‘sequence first, ask questions later’ approach, the portability of sequencing instruments such as the Oxford Nanopore Technologies (ONT) MinION makes this strategy a feasible option for almost-real-time studies in the field. Clinical microbiology with detection and characterization of pathogens in real time is indeed one of the fields that could be significantly revolutionized by such approaches in the near future. Loman continued describing how the ONT instrument played a key role in monitoring the outbreak of Ebola and in unraveling the molecular evolution of this virus. The technical limits of long-read technologies are, however, still some way ahead of us, with better protocols needed to keep DNA and RNA unfragmented, and technological improvements required to decrease sequencing errors.

Sonja Vernes (Max Planck Institute for Psycholinguistics, The Netherlands) shifted the focus from microbial genomics to bat genomics. Bats live for an exceptionally long time compared with what could be expected from their size, use sound to navigate in the dark and show high resistance against viruses. Insights into the genes and genetic mechanisms behind the unusual adaptations of bats might, for example, uncover the secrets to longer life spans, echolocation, and disease resistance. The Bat1K project (www.bat1k.com) aims to sequence and reconstruct the genomes of all approximately 1300 extant bat species. For such a massive genome-sequencing effort, the high quality of the reconstructed genomes will be guaranteed by the combination of short- and long-read sequencing technologies. This ensures the contiguity of the resulting assemblies owing to the long reads while maintaining a high single-nucleotide accuracy arising from the short reads. The project will present the first results by the end of 2019, when the genomes of representative species from 21 different bat families will be released.

## Single-cell sequencing

Single-cell sequencing is a technically challenging NGS-based approach to study the genomic and transcriptomic content of individual cells. It overcomes the traditional limitations of characterizing the heterogeneity of the micro-environment when DNA and RNA sequencing is performed on a mix of millions of cells. Sarah Teichmann (Wellcome Trust Sanger Institute, Hinxton, UK) presented exciting data from the Human Cell Atlas project (www.humancellatlas.org), which aims to build a comprehensive single-cell reference map of all human cell types. The potential of single-cell genomics was demonstrated by her work on fetal thymus tissues elucidating how the T-cell repertoire is formed. T cells were also targeted by Marlies Vanden Bempt (VIB-KU Leuven Center for Cancer Biology, Belgium) with her investigation into why cancer immunotherapy is effective in some patients and for some specific tumor types, but not for others. By studying cancerous cells at the single-cell level before and after immune-checkpoint inhibitor therapy, Vanden Bempt can assess what molecular and cellular mechanisms are contributing to resistance against these inhibitors. In the long run, these insights made possible by new sequencing-based technologies might lead to improved immunotherapy-based and personalized treatments.

## Metagenomics

Metagenomics is perhaps the field in which the current massive throughput typical of Illumina machines is the most relevant NGS feature, as retrieving more reads from the DNA pool extracted from an environmental sample translates into better detection of rare (micro)organisms. Despite the increasingly relevant role that metagenomic microbiome sequencing is playing in human biomedical fields, this technique was actually first applied more than 15 years ago on marine samples. Chris Bowler (IBENS, France) recapitulated the state-of-the-art in marine metagenomics by describing the Tara Oceans project—this enterprise has produced a huge data resource, with a catalog of approximately 150 million genes from 35,000 samples collected in more than 210 ocean stations across 20 biogeographic provinces. All the data are publicly available, and researchers worldwide have used them to profile the viruses, bacteria, archaea, and micro-Eukaryotes hidden in the massive marine layers of Earth. This is an outstanding example of how science in NGS-related fields can be advantaged by the community when analyzing large datasets that cannot be fully explored by any specific research group or consortium alone.

Julie Segre (National Human Genome Research Institute, NIH, USA) highlighted how metagenomics can be applied on a very different environment with minimal technological differences. She focused on the microbial diversity present on the human skin, which is a key component of the holobiont human assemblage. She described one of her most relevant breakthroughs on how the skin microbiome is individual specific but shaped by the local body biogeography. At the same time, however, the bacterial, fungal, and viral microbial communities of the skin remain largely stable over time despite the exposure of the skin to the external environment. Segre also pointed out the importance of strain-level resolution in microbiome research as different patient-specific strains have been found in the inflammatory skin disorder atopic dermatitis. Strain-level profiling from metagenomics was a common theme in the session, and this reflected how the field is moving towards very fine-grained analyses that are comparable in resolution to the genomic analyses performed with sequencing of microbial strains from pure culture. The genome-resolved analyses from the group of Segre have demonstrated how improvements in both NGS technologies (higher throughput, longer reads) and computational sequence analysis (e.g., better genome reconstructions) will be needed to fully exploit metagenomics in a biomedical setting.

## Emerging technologies

As the main theme of the RNGS conference revolved around advances in NGS, a session was organized in which emerging technologies in the field were presented. Among them, spatially resolved transcriptomics was introduced during the presentation of Mats Nilsson (Stockholm University, Sweden). He applied in situ sequencing at the single-cell level to build spatial maps of many thousands of cells in sections of mouse brains. Nilsson showed that data from this technique, when analyzed using 99 marker genes, allowed the investigators to identify the spatial organization of cell types across the brain and other tissues. Next, Andreas Tolias (Baylor College of Medicine, TX, USA) presented the Patch-seq approach to investigate the morphological phenotype, electrophysiological properties, and gene-expression patterns of neurons simultaneously by combining whole-cell patch-clamp recording, immunohistochemistry, and single-cell RNA sequencing. With this technology, Tolias and his team aim to eventually bridge the knowledge gap between neuronal structure and function on the road to decoding the algorithms of perceptual inference and decision-making used by the brain.

## Concluding remarks

One of the lessons learned from the first global sequencing efforts of the human genome is that advances in NGS and its applications are dependent on a strong interaction between the academic and industrial spheres. Although this report has focused on talks from representatives of academic groups only, the RNGS19 conference made this aspect central, with a variety of talks from companies at the forefront of the (next) sequencing revolution. Development of protocols from research groups that push new technologies from private companies beyond their original specifications is only one of the examples of successful interactions showcased at the conference. In all sessions, the results on specific applications, ranging from spatial transcriptomics to marine metagenomics, were accompanied by the feeling that new technologies are already enabling the next step in most of the NGS-based areas of research. From the complexity of new biotechnologies to the need for fine-tuned computational biological analyses, it is also clear that multidisciplinarity is key to moving the field forwards, and the RNGS19 conference was indeed a very welcome opportunity to initiate and foster interactions with researchers from different areas and to further revolutionize the sequencing field.

## Abbreviations

NGS: Next-generation sequencing
RNGS19: Revolutionizing Next-Generation Sequencing Conference 2019

